# Excitatory brain stimulation over the left dorsolateral prefrontal cortex enhances voluntary distraction in depressed patients

**DOI:** 10.1017/S0033291723000028

**Published:** 2023-10

**Authors:** Sijin Li, Jingxu Chen, Kexiang Gao, Feng Xu, Dandan Zhang

**Affiliations:** 1 School of Psychology, Shenzhen University, Shenzhen 518060, China; 2 Psychiatry Research Center, Beijing Huilongguan Hospital, Beijing, 100096, China; 3 Shenzhen Yingchi Technology Co., Ltd., Shenzhen 518057, China; 4 Institute of Brain and Psychological Sciences, Sichuan Normal University, Chengdu 610066, China; 5 Shenzhen-Hong Kong Institute of Brain Science, Shenzhen 518060, China; 6 Magnetic Resonance Imaging (MRI) Center, Shenzhen University, Shenzhen 518060, China

**Keywords:** Distraction, dorsolateral prefrontal cortex, emotion regulation, major depressive disorder, repetitive transcranial magnetic stimulation, social pain

## Abstract

**Background:**

While implicit distraction could ameliorate negative feelings in patients with major depressive disorders (MDD), it remains unclear whether patients could benefit from explicit, voluntary distraction. Meanwhile, though the dorsolateral prefrontal cortex (DLPFC) is established as a crucial brain region involved in attentional control, the causal relationship between the DLPFC and voluntary distraction is unexplored in patients.

**Methods:**

Combing explicit distraction and transcranial magnetic stimulation (TMS), this study investigated whether TMS-activated DLPFC facilitates voluntary distraction in MDD patients. Eighty patients diagnosed with current MDD underwent either active (*n* = 40) or sham (*n* = 40) TMS sessions, followed by receiving negative social feedback from other patients, during which they were requied to use distraction strategy to down-regulate their painful feelings. Electroencephalogram was recorded during the task.

**Results:**

Both the subjective emotional rating and the amplitude of late positive potential showed that depressed patients successfully down-regulate their negative emotions via voluntary distraction, and the TMS-activated left DLPFC produced a larger benefit of emotion regulation compared to the sham TMS group. Results also revealed that while emotion regulation effect was negatively associated with depressive symptoms in the sham TMS group, this correlation was largely diminished when patients' left DLPFC was activated by TMS during the voluntary distraction.

**Conclusions:**

These findings demonstrated that distraction is valuable for emotion regulation in MDD patients and they could be beneficial in voluntary distraction by activating their left DLPFC using neural modulation techniques. This study has valuable implications for clinical treatement of emotional dysregulation in MDD patients.

## Introduction

Major depressive disorder (MDD) is one of the most prevalent, disabling, and burdensome mental disorders worldwide (Friedrich, 2017; GBD, 2018). According to the cognitive model of depression (Beck & Bredemeier, 2016; Disner, Beevers, Haigh, & Beck, 2011; Lau & Waters, 2017), negative biases in attention, interpretation, attribution, and memory would result in sustained states of downcast mood, which plays an imperative role in the development and maintenance of MDD. Furthermore, difficulties in recovering from negative experiences would exacerbate depressive symptoms, thus initiating a vicious loop between persistent depressive episodes and emotion dysregulation (see Joormann and Stanton, 2016 for a review). Indeed, the deficit of emotion regulation is such a well-known problem in MDD patients that a mass of evidence has revealed their difficulty in voluntarily selecting adaptive strategies for emotion recovery as well as impaired prefrontal control network responsible for emotion regulation (see Joormann & Stanton, 2016; Rive et al., 2013 for reviews). Thus, it's critical and urgent to find a proper method to help recover patients' capability of emotion regulation.

One potential and frequently investigated strategy is distraction, which is considered as the ‘first aid’ tool to rapidly attenuate negative mood (Shafir, Schwartz, Blechert, & Sheppes, 2015). Distraction is implemented via attention redeployment, namely, redirecting attention to non-negative portions of the original scene or unrelated neutral thoughts so as to block in-depth processing of emotional information (Gross, 2015; Sheppes et al., 2014). Neuroimaging studies have shown that distraction leads to reduced negative feelings and decreased activation in the affective generation brain regions, such as the amygdala and insula, in both healthy individuals and depressive patients (Fales et al., 2008; Joormann, Siemer, & Gotlib, 2007; McRae et al., 2010; Wolkenstein & Plewnia, 2013). Typically, Kanske, Heissler, Schönfelder, and Wessa (2012) required depressed patients to do arithmetic when they viewed emotional pictures and found the distractive arithmetic task improved patients' emotional ratings of negative pictures. In line with these findings, the attention bias modification (ABM) procedures has been proposed as a promising antidepressant therapy to alter negative attention bias in patients (Browning, Holmes, Charles, Cowen, & Harmer, 2012). For instance, Beevers, Clasen, Enock, and Schnyer (2015) applied the ABM training using a dot-probe task (i.e. implicitly directing the attention of MDD patients away from negative pictures and toward neutral stimuli), which found that the active training group exhibited significant alleviation in depressive symptoms compared to the placebo group. Consistently, neuroimaging evidence showed reduced amygdala activity during viewing negative pictures in MDD patients after they received the ABM training (Hilland et al., 2020). While these pioneering studies provided important insights on attention deployment for emotion regulation, most of them examined passive or implicit distraction (Hilland et al., 2020; Kanske et al., 2012); that is, individual's emotional experience alters unconsciously without explicit regulation goals or deliberate attention control (Braunstein, Gross, & Ochsner, 2017; Etkin, Büchel, & Gross, 2015; Gyurak, Gross, & Etkin, 2011). Thus, these findings could not robustly attribute the improved emotional response and/or neural reactivity to the implementation of the distraction strategy. In contrast to implicit distraction, the explicit form of distraction involves predefined and distinct regulation goals as well as controlled attentional deployment (Braunstein et al., 2017; Etkin et al., 2015). This raised the first issue of this study that whether explicit and voluntary distraction is a valueable method for modulating emotions in depressed patients. Could the regulation benefits observed in implicit distraction generalize to explicit distraction in patients? This study used a standard emotion regulation task (Zhao et al., 2021) to directly examine the effect of explicit distraction on down-regulating negative emotions in MDD patients.

According to the neural model of emotional processing proposed by Etkin et al. (2015), implicit and explicit emotional regulation have both overlapping and distinct neural basis. In general, while implicit regulation predominantly involves the medial prefrontal cortices (PFC) to modulate subcortical affective generation areas, explicit emotional regulation additionally needs the lateral PFC regions for goal maintenance and top-down cognitive control (see Braunstein et al., 2017; Frank et al., 2014; Morawetz, Bode, Derntl, & Heekeren, 2017 for reviews). More relevant to the interest of this study, neuroimaging studies in healthy people have demonstrated that explicit distraction involves a large area of brain cortices including the frontal, parietal, and cingulate networks to serve the goal of top-down modulating the neural activity in emotion generative regions such as the amygdala and insula (Dörfel et al., 2014; Ferri, Schmidt, Hajcak, & Canli, 2016). Among these brain regions, the dorsolateral prefrontal cortex (DLPFC) plays a prominent role in attention deployment and maintenance, which modulates the amygdala activation via the parietal and cingulate pathways (Kanske et al., 2012; Li et al., 2021; Park et al., 2019; Zhao et al., 2021). In particular, healthy individuals show increased activation in the bilateral DLPFC and decreased negative feelings when they are instructed to direct attention to non-emotional portions of unpleasant pictures (Ferri et al., 2016; Ferri, Schmidt, Hajcak, & Canli, 2013). Also, evidence on functional connectivity indicates that distraction is accompanied by intensified negative coupling between the DLPFC and amygdala (Kanske, Heissler, Schönfelder, Bongers, & Wessa, 2011). A recent study in our lab used transcranial magnetic stimulation (TMS) and found that participants with TMS-facilitated DLPFC showed more significant social pain relief via distraction, compared to reappraisal, strategy, suggesting a relatively specific role of the DLPFC in voluntary distraction (rather than reappraisal) (Zhao et al., 2021).

Of note, the DLPFC (especially its left part) has been shown structurally and functionally damaged in depressed patients, reflected by reduced gray matter volume (Li et al., 2010; Sacher et al., 2012) and abnormal neural activation (Donofry, Roecklein, Wildes, Miller, & Erickson, 2016; Rive et al., 2013). Clinically, the left DLPFC is well recognized as a brain target for neuromodulation intervention during antidepressant treatment; using TMS to modulate and activate the left DLPFC has been proved to relieve depressive symptoms effectively in a considerable number of treatment-resistant MDD patients (Chen et al., 2013; George et al., 2010; Lefaucheur et al., 2020). Regarding to attention allocation that is relevant to distraction, previous studies found that MDD patients showed negative attention bias in the distracter inhibition task, reflected by increased amygdala response and reduced recruitment of the DLPFC in response to negative emotional distracters (Fales et al., 2008). Intriguingly, this negative attention bias in MDD patients was ameliorated by implementing anodal transcranial direct current stimulation (tDCS) on the left DLPFC, suggesting that facilitating the DLPFC could enhance the attentional control capability in patients (Wolkenstein & Plewnia, 2013). However, it has still been unexplored regarding the causal role of the left DLPFC in voluntary distraction in MDD patients.

The present study aimed to investigate whether TMS-activated left DLPFC could facilitate down-regulating negative emotions via voluntary distraction in patients with current MDD. We evaluated the distraction process in social scenarios because our previous studies showed that evoking and regulating painful social emotions enhances study power (He, Liu, Zhao, Elliott, & Zhang, 2020a; He et al., 2020b). In particular, adverse interpersonal events are highly self-relevant and often compose chief complaints of patients when they seek psychiatric treatment (Nasso, Vanderhasselt, Schettino, & De Raedt, 2022; Rappaport & Barch, 2020). Therefore, social pain would enhance their motivation for emotion regulation, compared to non-social negative emotions elicited by such as the International Affective Picture System (IAPS; Lang, Bradley, & Cuthbert, 2005). In line with this idea, we used an adapted version of the social judgment task (Somerville, Heatherton, & Kelley, 2006) to induce social pain in this study, during which MDD patients received negative social feedback from other patients and were informed to down-regulate their negative feelings using distraction strategy. Consistent with our previous studies (He et al., 2020b; Li et al., 2022; Zhao et al., 2021), we mainly examined two dependent variables. The first one was the subjective emotional rating, which has been widely used to assess emotional experiences (Mauss & Robinson, 2009). The second variable was the parietal late positive potential (LPP) amplitude, which is a sensitive electrophysiological marker of emotion reactivity (Kennedy & Montreuil, 2021). Many previous studies have revealed that down-regulating negative emotions via distraction could reliably reduce both negative feelings and LPP amplitudes (Shafir et al., 2015; Thiruchselvam, Blechert, Sheppes, Rydstrom, & Gross, 2011; Zhao et al., 2021). Considering the critical role of the DLPFC on distraction in healthy people, we expected in this study that TMS-facilitated DLPFC would result in attenuated negative emotional rating as well as reduced LPP amplitudes in MDD patients (compared to the patients in the sham TMS group) during explicit distraction.

## Methods

### Participants

During the experiment design, we conducted a prior power analysis using G*Power 3.1.7 (*F* tests, ANOVA: repeated measures, within-between interaction) based on the effect size (averaged 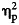 = 0.106) reported in our previous, related TMS study (He et al., 2020a; Li et al., 2022; Zhao et al., 2021). According to the result of this power analysis, 20 participants in total would ensure 95% statistical power. However, 10 participants per group are such a small sample size in present-day neuroscience studies. Thus, we finally decided to include 40 participants per TMS group, which ensured a statistical power near 100%.

As a result, a total of 80 patients were recruited from Beijing Huilongguan Hospital. They were diagnosed with a current major depressive episode according to the Diagnostic and Statistical Manual (DSM-IV; American Psychiatric Association, 1994). The diagnosis was based on a structured clinical interview for DSM-IV (SCID-I/P W/PSY SCREEN; First, Spitzer, Gibbon, and Williams, 2002). Exclusion criteria were current or lifetime neurological disorders and any comorbid Axis I disorder. At the time of the experiment, patients were either in their first-episode depression (*n* = 53) or receiving medication [antidepressants (*n* = 3), antipsychotics (*n* = 5), and both antidepressants & antipsychotics (*n* = 19)].

Patients were randomly assigned to the active (real) or sham TMS group. They completed five questionnaires on the day of the experiment: (1) the Beck Depression Inventory Second Edition (BDI-II; Beck, Steer, and Brown, 1996), (2) the Trait form of Spielberger's State-Trait Anxiety Inventory (STAI-T; Spielberger, Gorsuch, Lushene, Vagg, and Jacobs, 1983), (3) the Rejection Sensitivity Questionnaire (RSQ; Downey and Feldman, 1996), (4) the Liebowitz Social Anxiety Scale (LSAS; Liebowitz, 1987), and (5) the distraction subscale of the Thought Control Questionnaire (TCQ-D; Wells and Davies, 1994). As shown in Table 1, no significant difference was found between the two TMS groups with respect to demographical characteristics, age of illness onset, duration of illness, number of episodes, and medication use.

**Table 1.** Demographical characteristics of the two TMS groups (*M* ± s.e.)

Items	Active group (*n* = 40)	Sham group (*n* = 40)	Statistics^a^	
Gender (male/female)	11/29	8/32	χ^2^ = 0.621	*p* = 0.431
Handedness (right/left)	39/1	39/1	χ^2^ = 0.000	*p* = 1.000
Age (year)	28.9 ± 1.3	31.1 ± 1.6	*t* = −1.031	*p* = 0.306
Age at illness onset (years)	27.1 ± 1.4	28.4 ± 1.6	*t* = −0.614	*p* = 0.541
Duration of illness (month)	25.2 ± 5.4	31.7 ± 7.2	*t* = −0.720	*p* = 0.474
Number of episodes	0.3 ± 0.1	0.6 ± 0.9	*t* = −1.293	*p* = 0.200
Medications (A/B/C/D)^b^	30/1/1/8	23/2/4/11	χ^2^ = 3.532	*p* = 0.317
BDI-II	26.6 ± 1.1	26.6 ± 1.4	*t* = 0.000	*p* = 1.000
STAI-T	60.3 ± 1.3	61.0 ± 1.4	*t* = −0.319	*p* = 0.750
RSQ	12.7 ± 0.6	11.6 ± 0.5	*t* = 1.479	*p* = 0.143
LSAS	64.6 ± 5.5	65.2 ± 4.5	*t* = −0.095	*p* = 0.925
TCQ-D	12.2 ± 0.4	12.5 ± 0.4	*t* = −0.527	*p* = 0.599

BDI-II, the Beck Depression Inventory Second Edition; STAI-T, the Trait form of Spielberger's State-Trait Anxiety Inventory; RSQ, the Rejection Sensitivity Questionnaire; LSAS, the Liebowitz Social Anxiety Scale; TCQ-D, the distraction subscale of the Thought Control Questionnaire.

a χ^2^ test was performed on categorical variables. Independent samples *t* test (two-tailed) was performed on continuous variables.

b Number of MDD patients. A, no medication; B, antidepressants; C, antipsychotics; D, both antidepressants & antipsychotics.

Written informed consent was obtained prior to the experiment. The study was approved by the Ethics Committee of Beijing Huilongguan Hospital (approval number: 2020-38-research). We provided a free psychological consultation to each participant immediately after the experiment. The psychological consultation was conducted by an experienced psychiatrist (J. Chen) and lasted for at least one hour per patient (67.8 ± 8.3 min) for individualized treatment and rehabilitation suggestions. At the end of the consultation, all of the participant were in good moods and did not report any physical or emotional discomfort caused by the experimental manipulation.

### Experimental design and materials

The study was a 2 (*TMS group*: active *v.* sham) × 2 (*regulation type*: view *v.* distraction) mixed design. The within-subject factor was *regulation type* and the between-subject factor was *TMS group*.

During the task, we used 80 identity photos of adults (40 men and 40 women) who exhibited neutral facial expressions and appeared at the same ages as the patients (*M* ± s.e. = 28.9 ± 1.3, range = 18~56 years old). All photos were standardized in background color (white), resolution, and brightness. According to the ratings of these photos by another group of healthy adults (Xie, Hu, Mo, & Zhang, 2021), we assigned these photos into view and distraction conditions while their attractiveness and favorability were counterbalanced between conditions (*F*s < 1).

### Experimental procedure

**Fig. 1.**
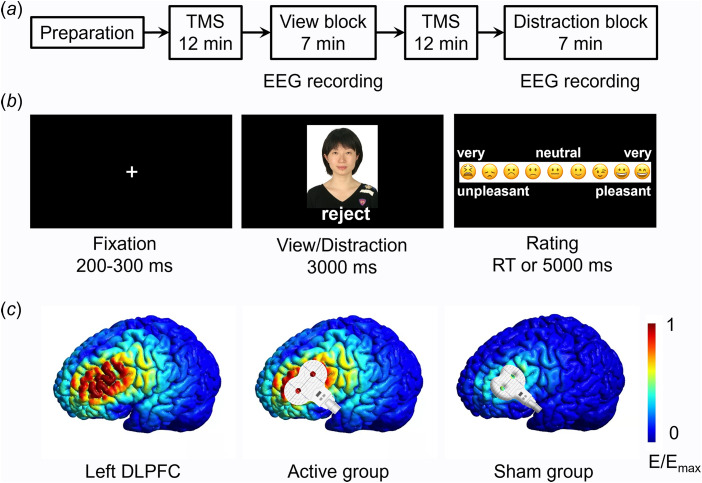
Experimental procedures. a, The procedure of the whole experiment. EEG data were recorded during the view block and the distraction block. b, Illustration of one trial. Here the person (i.e. an unfamiliar depressed patient) shown in the photo gave social feedback of ‘reject’ to the participant. Due to copyright, the person in the photo is replaced by one of the authors of this study (D.Z.). The event-related potential (ERP) was time-locked to the onset of social feedback. c, Illustration of TMS electric fields of the two TMS groups (active and sham). The coil was placed tangentially on the scalp in the active group and was placed at a 45° angle to the scalp in the sham group. The color represents the electric field strength, scaled from 0 (blue) to the individual maximums (red). DLPFC means the dorsolateral prefrontal cortex.

The experiment included a preparation phase, two TMS sessions, and two task blocks. Each task block was preceded by a TMS session (Fig. 1a).

The experiment was prepared three to five days prior to the experimental task. Participants were informed that a cost-free group therapy program for emotional release would be conducted in the upcoming week. The therapy has been proved to be effective in relieving unpleasant emotions and stress. It required five to eight participants to compose a group and share their physical and mental states during the therapy. Accordingly, participants were required to provide one identity photo of themselves and were told that their photos would be evaluated by several unfamiliar depressed patients, who would decide whether to accept them as group members during the therapy based on their first impression of participants' photos.

During the view block, participants were instructed to attend to the results of the first impression evaluation (i.e. social feedback) provided by other MDD patients (Fig. 1b). They should pay attention to both the feedback and the feedback sender and react naturally during emotional rating in each trial. During the distraction block, participants were instructed to attend to non-negative information on the screen so as to down-regulate their unpleasant feelings. For example, they were suggested to attend to the configuration characteristics or one part (e.g. eyes, mouth, etc.) of the feedback sender's face. Before the formal task, all the participants were trained to properly and accurately use the distraction strategy, that is to say, they were asked to verbally report their thoughts during the emotion regulation in practice trials to ensure that they understood the distraction method. To avoid any carry-over effects caused by the distraction instruction, the two blocks had a fixed order, i.e., the view block always ran first, followed by the distraction block (see also He et al., 2018; Krompinger, Moser, & Simons, 2008; Troy, Shallcross, & Mauss, 2013; Zhao et al., 2021).

In each block, the 40 photos of peers together with their feedback were presented in a random order. Among the 40 stimuli, 10 patients gave ‘accept’ feedback (indicating their willingness to be assigned to the same therapy group with the participant) while the other 30 patients gave ‘reject’ feedback (indicating their unwillingness to compose a group with the participant). The 30 negative feedback was used to induce social pain and thus composed the 30 valid trials in each block. As shown in Fig. 1b, each trial began with a fixation, followed by the presentation of a photo-feedback combination for 3000 ms, during which participants were required to either view passively (in the view block) or to regulate their emotion (in the distraction block). Then participants were asked to report how they felt on a continuous scale of 0 to 1 (‘0’ for very unpleasant and ‘1’ for very pleasant) by clicking the left button on the mouse within 5000 ms. At the end of each trial, a blank screen appeared for 1000 ms.

### Repetitive transcranial magnetic stimulation (rTMS)

This study used offline, instead of online, TMS to reduce possible confounding factors that may impact participants' task performances (e.g. uncomfortable scalp sensations) or EEG recordings (TMS-induced electric artifacts). The TMS target was the left DLPFC. A figure-eight-shaped coil was connected to the magnetic stimulator (M-100 Ultimate; Yingchi, Shenzhen, China). The location of the coil was determined with reference to the International 10/20 electroencephalogram system, i.e., the F3 site (Beam, Borckardt, Reeves, & George, 2009; Herwig, Satrapi, & Schönfeldt-Lecuona, 2003; Xie, Chen, Lin, Hu, & Zhang, 2020). Each participant's resting motor threshold (rMT) was measured from their motor cortex (the C3 site), with the intensity being defined as 50% of the pulses that reliably produced finger twitches. Among the 40 participants in the active TMS group, we could find the hot-spot (the finger motor cortex) in 35 of them (87.5%) within 5 min. For these participants, stimulus magnetic pulses were delivered at 90% intensity of the rMT. For the other 5 participants in the active TMS group and the 40 ones in the sham group, stimulus magnetic pulses were delivered at 90% intensity of the maximal intensity that can be tolerated. For individuals in the active TMS group, the coil was placed tangentially on the scalp so that most of the magnetic field lines could go through the scalp and reach the left DLPFC. For participants in the sham group, the coil was placed at a 45° angle to the scalp so very limited magnetic field lines could reach the brain (see Kimbrell et al., 1999; Xie et al., 2020). The rTMS was applied at 10 Hz with a duration of 12 min per session, which was proved to increase excitability in the targeted area (Dayan, Censor, Buch, Sandrini, & Cohen, 2013). There were two sessions of rTMS, each prior to the view or distraction block. Each 12 min session contained 24 trains, with each train lasting for 4 s (a total of 960 pulses) and being separated by inter-train intervals of 26 s. The simulated electric field induced by TMS is illustrated in Fig. 1c (SimNIBS, www.simnibs.org, Thielscher, Antunes, and Saturnino, 2015).

All the participants were told before the experiment that the rTMS device is safe and has little chance to produce side effect according to previous statistical results based on a huge number of samples. Also, they were informed about the rationale, stimulation procedure, and possible feelings of TMS before the experiment. None of them terminated the experiment because of uncomfortable sensations induced by TMS.

### EEG recordings and analysis

EEG data were recorded during the view block and the distraction block using a 32-channel amplifier (Brain Products, Gilching, Germany), with a sampling frequency of 250 Hz. Electrode impedances were kept below 10 kΩ. The reference electrode was placed at the TP9.

The ERP recording and analysis were designed especially for the LPP (He et al., 2020b; Li et al., 2022; Zhao et al., 2021). The electrodes and time window for the measurement of LPP amplitudes were decided prior to analysis. Data were first re-referenced to the average of the bilateral mastoids, followed by filtering using a 0.1~10 Hz band-pass filter with a slope of 24 dB/oct. The filtered data were segmented beginning 200 ms prior to the onset of the feedback and lasting for 3 s. The baseline-correction was based on the 200 ms pre-stimulus time window. We measured the LPP as the average amplitude across the electrode sites at and around Pz (P3, P4, Pz, CP1, CP2). The time window for the LPP amplitude began at the end of the typical P3 time window (500 ms) and lasted for the entire emotion regulation period (500~3000 ms post feedback onset; see also Hajcak & Nieuwenhuis, 2006; Paul, Simon, Endrass, & Kathmann, 2016; Zhao et al., 2021).

The EEG data of two participants were corrupted due to technical problems. As a result, 78 datasets (39 in the active group and 39 in the sham group) were involved in the following analyses.

## Results

### Subjective emotional rating

Statistical analysis was performed using SPSS Statistics 20.0 (IBM, Somers, USA). First, paired samples *t* test was performed between the emotional ratings following positive and negative feedback. It revealed that participants felt much more negative after receiving ‘reject’ (0.481 ± 0.008, *M* ± s.e.) than ‘accept’ (0.566 ± 0.008) feedback (*t* = −8.1, *p* < 0.001), indicating a successful manipulation of social pain induction.

**Fig. 2.**
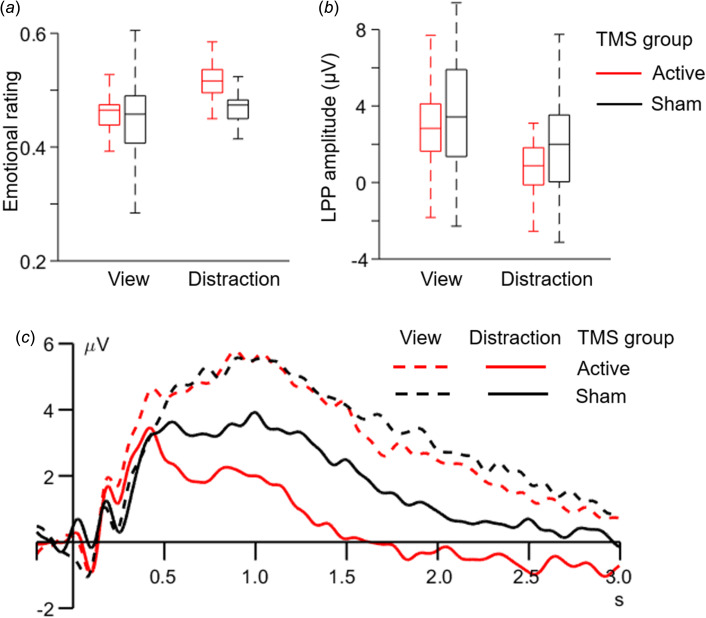
Illustration of main results. a, Subjective emotional rating. Participants reported their emotional feelings on a continuous scale (0 for very unpleasant, 1 for very pleasant). The interaction between *TMS group* and *regulation type* was significant. b, Mean amplitudes of the LPP component. The interaction between *TMS group* and *regulation type* was significant. Here we use boxplots to show the data. On each box, the central line indicates the median, and the bottom and top edges of the box indicate the 25th and 75th percentiles, respectively. The whiskers indicate 1.5 times the interquartile range from the ends of the box. c. ERP waveforms containing the LPP. The ERP data were averaged across electrodes of Pz, P3, P4, CP1, and CP2.

Then a two-factor mixed-design ANOVA was performed on subjective ratings, with *regulation type* (view or distraction) as the within-subject factor and *TMS group* (active or sham) as the between-subject factor. Greenhouse–Geisser correction was used for testing model assumptions. The main effect of the *regulation type* was found to be significant (*F*_(1,76)_ = 52.3, *p* < 0.001, 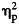 = 0.407): participants reported more positive feelings in the distraction block (0.503 ± 0.008) than the passive view block (0.459 ± 0.009). The main effect of *TMS group* was not significant (*F*_(1,76)_ = 0.8, *p* = 0.374, 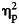 = 0.010; active = 0.489 ± 0.012, sham = 0.474 ± 0.012). The two-way interaction between *TMS group* × *regulation type* was significant (*F*_(1,76)_ = 8.6, *p* = 0.004, 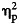 = 0.102; Figure 2a). Simple effect analysis showed that while the two groups did not differ in emotional rating during the view block (*F*_(1,76)_ = 0.0, *p* = 0.859, 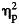 = 0.000; active = 0.458 ± 0.013, sham = 0.461 ± 0.013), the emotional rating tended to be more positive in the active TMS group (0.520 ± 0.012) compared to that in the sham group (0.487 ±0.012) during the distraction block (*F*_(1,76)_ = 3.8, *p* = 0.054, 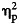 = 0.048; marginal significance). Another direction of simple effect analysis indicated that although the patients in both the groups reported more positive feelings in the distraction block than the view block, this emotion regulation effect was more distinct in the active (*F*_(1,76)_ = 51.7, *p* < 0.001, 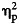 = 0.405) compared to the sham group (*F*_(1,76)_ = 9.2, *p* = 0.003, 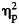 = 0.108). Detailed statistics of simple effect analysis are listed in Table 2.

**Table 2.** Statistical results of the two-way interaction between *TMS group* and *regulation type*

Dependent variables	Interaction			Pairwise comparison				
	*F*	*p*	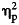	Condition	*M* *±* s.e.	*F*	*p*	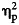
Emotional rating	8.6	0.004	0.102	View: sham group View: active group	0.461 ± 0.013 0.458 ± 0.013	0.0	0.859	0.000
				Distraction: sham group Distraction: active group	0.487 ± 0.012 0.520 ± 0.012	3.8	0.054	0.048
				Sham group: view Sham group: distraction	0.461 ± 0.013 0.487 ± 0.012	9.2	0.003	0.108
				Active group: view Active group: distraction	0.458 ± 0.013 0.520 ± 0.012	51.7	< 0.001	0.405
LPP amplitude	4.3	0.042	0.053	View: sham group View: active group	3.46 ± 0.42 *μ*V 3.15 ± 0.42 *μ*V	0.3	0.609	0.003
				Distraction: sham group Distraction: active group	1.80 ± 0.43 *μ*V 0.35 ± 0.43 *μ*V	5.6	0.021	0.068
				Sham group: view Sham group: distraction	3.46 ± 0.42 *μ*V 1.80 ± 0.43 *μ*V	18.4	< 0.001	0.195
				Active group: view Active group: distraction	3.15 ± 0.42 *μ*V 0.35 ± 0.43 *μ*V	52.0	< 0.001	0.406

**Fig. 3.**
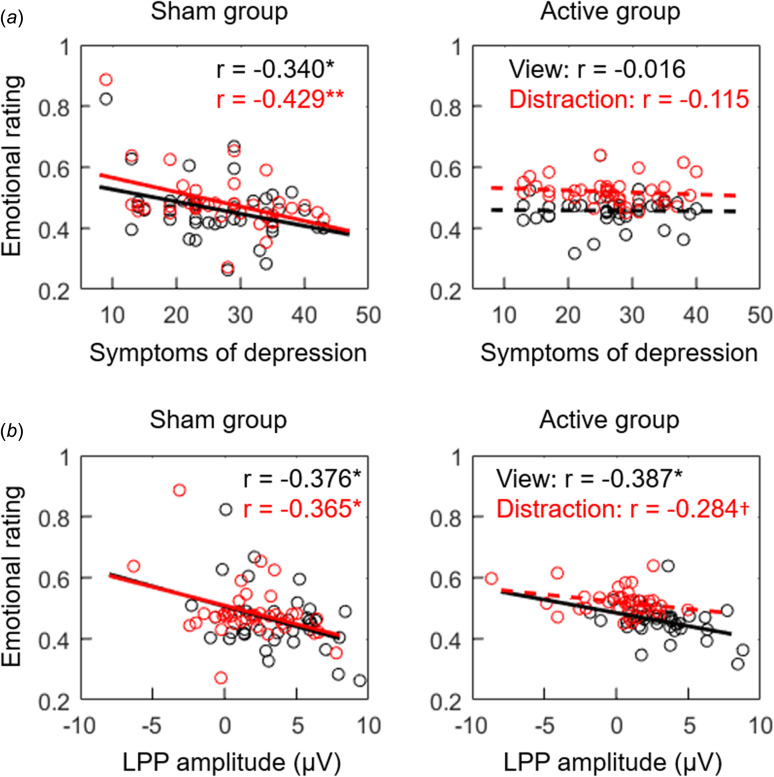
Correlation findings. a, The correlation between subjective emotional rating and symptoms of depression (measured by the BDI-II). b, The correlation between subjective emotional rating and LPP amplitude. Solid lines show significant fitting lines while dashed lines show the fitting lines that were not significant. The black and red circles denote individual data in the view and distraction blocks, respectively. †*p* < 0.10, *p < 0.05, and **p < 0.01.

Two-tailed Pearson's correlation analysis showed that while the subjective emotional rating was negatively correlated with the symptoms of depression (measured by the BDI-II) in the sham TMS group (view: *r* = −0.340, *p* = 0.034; distraction: *r* = −0.429, *p* = 0.006; Figure 3a), this correlation was not significant in the active TMS group (view: *r* = −0.016, *p* = 0.923; distraction: *r* = −0.115, *p* = 0.485).

### LPP amplitude

In line with the analysis of subjective rating, a two-factor mixed design ANOVA (Greenhouse–Geisser corrected) was also performed on LPP amplitudes (Fig. 2c). The main effect of *regulation type* was found to be significant (*F*_(1,76)_ = 66.1, *p* < 0.001, 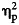 = 0.465; view = 3.30 ± 0.30 *μ*V, distraction = 1.07 ± 0.31 *μ*V). The main effect of *TMS group* was not significant (*F*_(1,76)_ = 2.6, *p* *=* 0.109, 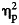 = 0.033; active = 1.75 ± 0.38 *μ*V, sham = 2.63 ± 0.38 *μ*V). The two-way interaction between *TMS group* × *regulation type* was significant (*F*_(1,76)_ = 4.3, *p* *=* 0.042, 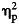 = 0.053; Fig. 2b). Simple effect analysis showed that while the two groups did not differ in LPP amplitudes during the view block (*F*_(1,76)_ = 0.3, *p* *=* 0.609, 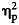 = 0.003; active = 3.15 ± 0.42 *μ*V, sham = 3.46 ± 0.42 *μ*V), the LPP was smaller in the active TMS group (0.35 ± 0.43 *μ*V) compared to that in the sham group (1.80 ± 0.43 *μ*V) during the distraction block (*F*_(1,76)_ = 5.6, *p* *=* 0.021, 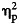 = 0.068). Another direction of simple effect analysis indicated that although the patients in both the TMS groups exhibited lower LPP amplitudes in the distraction block than in the view block, this emotion regulation effect was more distinct in the active (*F*_(1,76)_ = 52.0, *p* < 0.001, 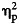 = 0.406) compared to the sham group (*F*_(1,76)_ = 18.4, *p* < 0.001, 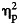 = 0.195). Detailed statistics of simple effect analysis are listed in Table 2.

Finally, Pearson's correlation analysis was conducted between LPP amplitudes and subjective emotional rating. In general, the two indices were negatively correlated (or had a trend of negative correlation) in both the sham TMS group (view: *r* = −0.376, *p* = 0.018; distraction: *r* = −0.365, *p* = 0.022) and the active TMS group (view: *r* = −0.387, *p* = 0.015; distraction: *r* = −0.284, *p* = 0.080; Fig. 3b).

## Discussion

Using TMS combined with the standardized explicit distraction introduction, this study investigated the causal role of the left DLPFC on voluntary distraction in patients with MDD. We found that voluntary distraction is an applicable strategy for depressed patients which helps successfully down-regulate their negative emotions, and that excitatory TMS over the left DLPFC facilitated voluntary distraction, indicating a potential translational value of improving everyday mood in MDD patients.

In line with our hypothesis, the result shows that patients diagnosed with current MDD are capable of employing the distraction strategy to diminish social pain evoked by negative social feedback, as evidenced by both the self-reported and electrophysiological indices. The findings align well with previous studies showing that diverting attention away from emotional salient aspects leads to attenuated unpleasant experiences in MDD patients (Kanske et al., 2012; Kovacs et al., 2015; Smoski, LaBar, & Steffens, 2014). One critical difference between the previous and present studies was that the former investigated implicit distraction effects (e.g. Kanske et al., 2012) while we explored explicit distraction. Thus, this study directly demonstrated the benefits of voluntary distraction in MDD patients. Moreover, considering that interpersonal and social problems compose the main causes of MDD (Disner et al., 2011; Rappaport & Barch, 2020), this study induced social pain instead of non-social negative emotion so to provide a more similar affective experience of patients in their daily life. While impaired emotion processing has been often found in depressed patients when they experienced social pain, e.g., lowered pain thresholds (Ehnvall et al., 2014; Jobst et al., 2015), intensified unpleasant feelings (Bauriedl-Schmidt et al., 2017; Seidl et al., 2020), and difficulties in affective recovering (Hsu et al., 2015), the current finding demonstrates that MDD patients retain the capability of voluntarily diverting attention away from negative aspects of events during emotion regulation.

The most important finding is the neural modulation effect, that is, the TMS-activated left DLPFC produced a larger benefit of voluntary distraction compared to the sham TMS group. The result confirms our previous finding that the DLPFC is essential and plays a causal role on voluntary distraction (Zhao et al., 2021). The DLPFC has been a well-known attentional control brain region as revealed by numerous neuroimaging studies. For instance, this region is involved in emotional distraction (Iordan, Dolcos, & Dolcos, 2019), directed attention (Ferri et al., 2013), and selective attention (Browning, Holmes, Murphy, Goodwin, & Harmer, 2010). Causal evidence also suggested that anodal tDCS on the left DLPFC could improve cognitive inhibition of emotional distracters in MDD patients (Wolkenstein & Plewnia, 2013); also, cathodal tDCS on the left DLPFC led to depression-like attention bias in healthy individuals (Wolkenstein, Zeiller, Kanske, & Plewnia, 2014). A recent study further found that excitatory TMS over the left DLPFC intensified the negative connectivity between the DLPFC and amygdala in MDD patients (Eshel et al., 2020). Accordingly, the TMS benefit observed in this study might be due to the intensified DLPFC-amygdala connectivity that helps patients engage in top-down control of neural responses in subcortical emotion generative networks. Of note, the observed neural modulation benefit of TMS was evidenced by both the self-reported emotional rating and electrophysiological index of LPP amplitude. Also, the two indices of emotion regulation effect were well correlated in all the conditions across the two groups (see also Hajcak & Nieuwenhuis, 2006; Li et al., 2022; Yuan et al., 2015; Zhao et al., 2021), which provide convergent evidence for the role of DLPFC in voluntary distraction.

Our findings have valuable implications for clinical treating emotional dysregulation in patients. While the left DLPFC is already a well-established cortical target of TMS intervention for relieving depressive symptoms (George et al., 2010; Lefaucheur et al., 2020), this study provides a clear empirical rationale for improving emotion regulation capabilities by activating the left DLPFC of patients. In this study, it is found that the self-reported emotional rating was negatively associated with the severity of depressive symptoms (see also Erk et al., 2010; Troy, Wilhelm, Shallcross, & Mauss, 2010), indicating that depressive symptoms indeed impair the capability of emotion regulation. However, this correlation was largely diminished when the left DLPFC was activated by TMS during the voluntary distraction, suggesting the potential beneficial changes of emotion regulation in patients treated with TMS. Considering that attention modification training has been proved valuable for depressed patients (Browning et al., 2012; Dai, Hu, & Feng, 2019; Yang, Zhang, Ding, & Xiao, 2016), we suggest future studies to explore the protocol that combines attention modulation training (i.e. distract attention from negative stimuli) and TMS therapy (target on the left DLPFC) to reach an optimal therapeutic effect.

Some limitations should be noticed when interpreting the current findings. First, we used a fixed order of the conditions (first baseline and then distraction) to avoid a potential influence of explicit instruction on the baseline condition. Although this design has been proved to produce insignificant confounding effects (e.g. habituation or fatigue) in relevant studies (Yuan et al., 2022), we suggest future studies to use random or counterbalanced order (e.g. Nguyen, Zhou, Potter, Zou, & Zhang, 2019; Thiruchselvam et al., 2011) to verify the current finding. Second, we only examined the immediate effect of TMS-facillitated distraction process. Future effort should be made to translate the short-term effect to long-term emotional benefits in patients by using, for example, multi-session TMS protocals. Meanwhile, cognitive reappraisal is another efficient strategy to regulation emotion (Ochsner, Silvers, & Buhle, 2012). Examining and comparing the effects of distraction and reappraisal in a single study (see Dörfel et al., 2014; Zhao et al., 2021 for studies in healthy populations) might provide comprehensive knowledge on voluntary emotion regulation in MDD patients. Third, considering that the DLPFC engages in not only voluntary distraction but also reappraisal and other cognitive control processes (Morawetz et al., 2017), we could not exclude the possibility that the current finding in the distraction condition was influenced by cognitive processes in addition to distraction. Post-hoc interviews (e.g. Kim and Hamann, 2007) and eye-tracking devices (e.g. Ferri et al., 2013) are suggested in future studies to confirm the attention deployment procedure during the task.

In conclusion, both the behavioral and electrophysiological indices demonstrate that (1) distraction is a valuable method for emotion regulation in MDD patients, (2) the left DLPFC is an essential cortical region involved in voluntary distraction in patients, and (3) patients could be beneficial in emotion regulation by activating their left DLPFC using neural modulation devices such as TMS.

## Data Availability

The data and code of this study would be available upon reasonable request and with approval of the School of Psychology, Shenzhen University. More information on making this request can be obtained from the corresponding author, D. Zhang (zhangdd05@gmail.com).
